# Microbial Synthesis and Evaluation of Fungistatic Activity of 3-Butyl-3-hydroxyphthalide, the Mammalian Metabolite of 3-*n*-Butylidenephthalide

**DOI:** 10.3390/ijms22147600

**Published:** 2021-07-15

**Authors:** Joanna Gach, Teresa Olejniczak, Piotr Krężel, Filip Boratyński

**Affiliations:** Department of Chemistry, Wrocław University of Environmental and Life Sciences, Norwida 25, 50-375 Wrocław, Poland; piotr.krezel@outlook.com (P.K.); filip.boratynski@upwr.edu.pl (F.B.)

**Keywords:** biotransformations, Apiaceae, bioactivity, phthalides, lipophilicity, lactones, antifungal activity

## Abstract

Phthalides are bioactive compounds that naturally occur in the family Apiaceae. Considering their potentially versatile applications, it is desirable to determine their physical properties, activity and metabolic pathways. This study aimed to examine the utility of whole-cell biocatalysts for obtaining 3-butyl-3-hydroxyphthalide, which is the metabolite formulated during mammalian metabolism of 3-*n*-butylidenephthalide. We performed transformations using 10 strains of fungi, five of which efficiently produced 3-butyl-3-hydroxyphthalide. The product yield, determined by high-performance liquid chromatography, reached 97.6% when *Aspergillus candidus* AM 386 was used as the biocatalyst. Increasing the scale of the process resulted in isolation yields of 29–45% after purification via reversed-phase thin layer chromatography, depending on the strain of the microorganism used. We proposed different mechanisms for product formation; however, hydration of 3-*n*-butylidenephthalide seems to be the most probable. Additionally, all phthalides were tested against clinical strains of *Candida albicans* using the microdilution method. Two phthalides showed a minimum inhibitory concentration, required to inhibit the growth of 50% of organisms, below 50 µg/mL. The 3-*n*-butylidenephthalide metabolite was generally inactive, and this feature in combination with its low lipophilicity suggests its involvement in the detoxification pathway. The log *P* value of tested compounds was in the range of 2.09–3.38.

## 1. Introduction

Phthalides are a group of bioactive secondary metabolites that are found mainly in plants from the family Apiaceae, such as *Angelica acutiloba, Angelica sinensis, Apium graveolens*, *Cnidium officinale, Levisticum officinale* and *Ligusticum porteri* [1,2,3,4,5,6]. These compounds have been widely tested for their pharmacological properties [7,8,9,10,11]. 3-*n*-butylphthalide has been authorized for treating brain ischemia in China [7]. Moreover, the analog of this compound, 3-*n*-butylidenephthalide, showed a positive effect on the cardiovascular system, inhibiting angiogenesis in vitro, ex vivo and in vivo in animal models [8]. Furthermore, 3-*n*-butylidenephthalide also exhibited neuroprotective activity by reducing secreted proinflammatory molecules in rat brain glial cells, thus increasing the lifespan of mice with amyotrophic lateral sclerosis [9,10]. A study also reported its antihyperglycemic activity in an animal model [11].

Phthalides have also been examined as potential fungistatic agents. 3-*n*-butylidenephthalide appeared to be more active against dermatophytes than the antifungals itraconazole and ketoconazole [12]. Significantly, similarly structured 3-*n*-butylphthalide inhibited the growth of four clinical strains of *Candida albicans*, while the reference drug, fluconazole, was inactive at much higher concentrations [13]. *Candida* species are one of the most common causes of fungal infections worldwide. Even though these microorganisms are ubiquitous in humans, they can establish mucus membrane infections in cases of microbiome disturbances or even severe systemic candidiasis in immunocompromised patients [14,15,16]. The development of novel antifungals is necessitated by the rising resistance of *Candida* yeasts to antifungals, such as azoles, and drawbacks of these compounds, such as toxicity and possible drug-drug interactions [17,18,19,20]. Notably, approximately 80% of antifungal agents are not investigated owing to their adverse effects [20]. The possible pharmacological application of phthalides warrants the assessment of their metabolic pathways. It is crucial to evaluate the toxicity and bioavailability of novel treatments at the earliest possible stage in order to avoid problems during subsequent phases. For instance, compounds may undergo bioactivation to reactive hepatotoxic derivatives [21]. Thus, the products of phthalide metabolism must be studied extensively. The small amounts of drug metabolites tested in vivo may pose an issue with their detection [22]; therefore, the in vitro preparation of metabolites may allow for easier determination of their structure and facilitate further investigations. Microbial whole-cell transformation is a viable system utilized for this purpose; it enables the metabolite to be obtained in high quantities and at a feasible cost [23].

Fungi-mediated biotransformations allow production of compounds with high regio- and stereoselectivity through reduction, oxidation, epoxidation, hydrogenation, hydrolysis, amination, acylation, glucosylation, epoxidation, hydration, methylation and hydroxylation by cytochrome P450 enzymes, especially regarding steroid conversion [24,25,26]. Moreover, the valuable characteristic of a fungi-based approach is the complexity of their metabolic abilities, allowing for xenobiotic degradation that is partially reminiscent of mammalian metabolism [26].

Diao et al. reported that hydroxylation of 3-*n*-butylphthalide, a saturated analogue of 3-*n*-butylidenephthalide, was the main metabolic route in humans, and that this hydroxylation occurred on the side chain instead of the relatively stable benzene ring. Similarly, use of this substrate in the culture of *Cunninghamella blakesleana* ATCC9244 also yielded hydroxylated compounds, including hydroxy-3-butylphthalide [27]. Furthermore, Yan et al. reported the formation of this product during the metabolism of 3-*n*-butylidenephthalide in rats [28]. Nevertheless, 3-*n*-butylidenephthalide has not previously been transformed using microbial whole cells.

Lipophilicity, defined as the affinity of a compound towards nonpolar phase, is also significant for potential drug evaluation [29]. The log *P* parameter is linked to the absorption, distribution, metabolism and excretion processes [30,31]. It has been shown that lipophilicity that is too high is associated with undesirable properties, such as accumulation of the compound in the adipose tissue [32]. According to Lipinski’s rule of five, the log *P* value should not exceed 5 [33].

Research on phthalides and their metabolites should focus not only on determining their structure, but also on assessing their physical properties, activity and metabolic pathways by analyzing structural analogs. Therefore, our primary aim was to determine whether the fungi transform 3-*n*-butylidenephthalide to 3-butyl-3-hydroxyphthalide: the identical compound that is formed in small quantities during rat metabolism, precluding its isolation. Efficient synthesis of this metabolite using microorganisms would be an attractive alternative approach in comparison to chemical synthesis.

Moreover, we aimed to elucidate the mechanisms of the biotransformation process. Considering the possible pharmacological applications of this research, we also assessed the lipophilicity of the substrate and its metabolite and compared their inhibitory potential against four *Candida albicans* strains.

## 2. Results and Discussion

Screening biotransformations involving fungal strains were performed to select the most effective biocatalysts for producing 3-butyl-3-hydroxyphthalide (**2**). This approach allowed the resulting microorganisms to be used in scale-up processes to isolate the metabolite and confirm its structure by nuclear magnetic resonance (NMR) spectroscopy (Figures S1–S16, Supplementary Materials). Next, we evaluated the lipophilicity of compounds using reversed-phase high-performance liquid chromatography (RP-HPLC) and tested their antimicrobial properties against selected *Candida* yeasts. We also aimed to establish whether a metabolite with a different polarity also exhibits bioactive potential or is suggestive of xenobiotic detoxification.

### 2.1. Biotransformations of 3-n-Butylidenephthalide (1)

**Figure 1:**
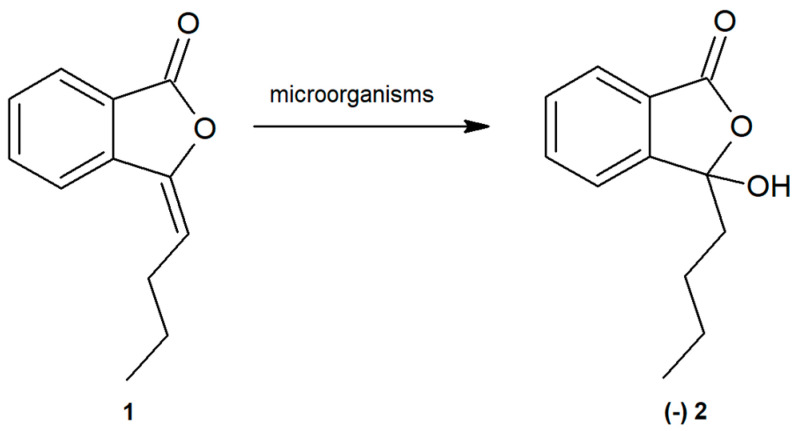
Biotransformation of 3-*n*-butylidenephthalide (**1**) to (-)-3-butyl-3-hydroxyphthalide (**2**) using whole fungal cells.

To obtain 3-butyl-3-hydroxyphthalide (**2**) from 3*-n*-butylidenephthalide (**1**), wholecells of fungi were used (Figure 1).

Initially, screening-scale biotransformation of 3-*n*-butylidenephthalide (**1**) was conducted using 10 fungal strains, which were analyzed for their catalytic potential. These strains included *Absidia cylindrospora* AM 336, *Ascosphaera apis* AM 496, *Aspergillus candidus* AM 386, *Chaetomium indicum* AM 158, *Fusarium culmorum* AM 9, *Fusicoccum amygdali* AM 258, *Laetisporus sulphurens* AM 515, *Mucor spinosus* AM 398, *Penicillium chrysogeum* AM 112 and *Pycnidiella resinae* AM 50 (data not shown). Of these, the five strains that exhibited the most efficient product (**2**) formation were *Aspergillus candidus* AM 386, *Absidia cylindrospora AM* 336, *Mucor spinosus* AM 398, *Chaetomium indicum* AM 158 and *Pycnidiella resinae* AM 50. These five strains were chosen for further screening.

Biotransformation of 3-*n*-butylidenephthalide (**1**) as a mixture of (*E*) and (*Z*) isomers (9:1) at a concentration of 0.27 mg/mL was carried out for 14 days at 25 °C with shaking culture in Sabouraud medium. Samples were taken every 2 days, extracted with ethyl acetate and analyzed via thin layer chromatography (TLC) and HPLC (Figures S14–16, Supplementary Materials).

**Figure 2:**
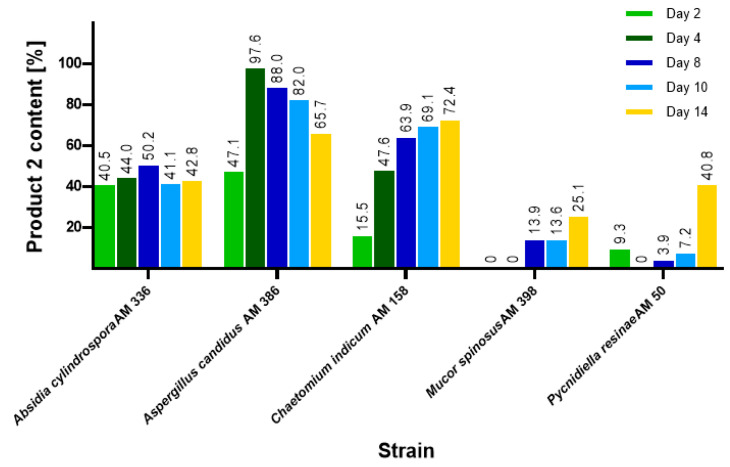
Content of product (**2**) during the screening-scale biotransformations of 3-*n*-butylidenephthalide (**1**) determined using a high-performance liquid chromatography-diode array detector.

**Figure 3:**
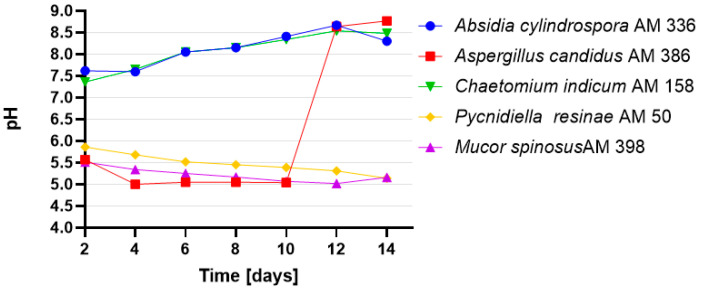
pH values of cultures during the screening-scale transformations of 3-*n*-butylidenephthalide (**1**).

The highest product (**2**) yield of 97.61% was obtained using *A. candidus* AM 386 on day 4, as determined by HPLC (Figure 2). However, in contrast to that in other strains, *A. candidus* AM 386 further metabolized compound (**2**) to unidentified derivatives. The other tested microorganisms, *A. cylindrospora* AM 336, *M. spinosus* AM 398, *C. indicum* AM 158 and *P. resinae* AM 50, produced yields between 25.13% and 72.40% by day 14. We also focused on the pH of the cultures over the course of the biotransformations (Figure 3). In cultures with *M. spinosus* AM 398 and *P. resinae* AM 50, the conditions were slightly acidic (pH 5–6), whereas the *A. cylindrospora* AM 336 and *C. indicum* AM 158 strains subtly alkalized the culture (pH 7.4–8.5). In the *A. candidus* AM 386 culture, the pH was in the range of 5.5–5.0 in the first few days but subsequently increased to 8.5. The sudden change in pH value presumably occurred due to the change in nutrient availability. During culture growth, depletion of the carbon source causes stress for the microbial cells, resulting in extracellular release of ammonia [34]. The stability of the compounds under extreme pH conditions were examined using a mixture of substrates and products.

**Figure 4:**
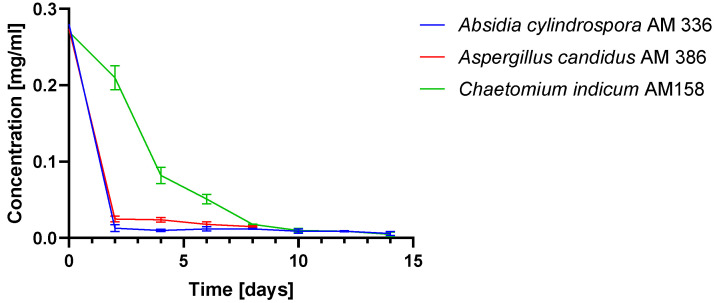
Change in 3-*n*-butylidenephthalide (**1**) concentration during scale-up biotransformation determined by gas chromatography.

In order to confirm the structure of compound (**2**) and determine its mechanism of action, we scaled up the biotransformation with the following strains: *A. candidus* AM 386, *A. cylindospora* AM 336 and *C.* *indicum* AM 158. We decided to use three strains on a preparative scale instead of focusing on only one of them, as the isolation yields of product (**2**) might have been diversified. This issue may result from different metabolite concentrations in particular strains, which are not visible during screening-scale transformation while the biotransformation is monitored by HPLC. We also sought to determine the stereoselectivity of the process for particular strains. Biotransformations were performed in 2000-mL shaken flasks with 3-*n*-butylidenephthalide (**1**) at a concentration of 150 mg/500 mL at 25 °C. The products were analyzed using gas chromatography (GC), HPLC and RP-TLC. All three microorganisms sufficiently transformed 3-*n*-butylidenephthalide (**1**) (Figure 4). The conversion of the substrate was slower in *C. indicum* AM 158, which is consistent with the results of the small-scale experiments. The biotransformations continued until day 14 with the exception of *A. candidus* AM 386, which was only continued to day 8 because of an observed decrease in product yield.

The pH of the culture media was in the range of 7–8.25 when using fungi from the genera *Chaetomium* and *Absidia*. Meanwhile, the pH of cultures of fungi from the genus *Aspergillus* was acidic and remained stable at pH 5 during biotransformation. Monitoring the pH value allowed us to determine whether it was necessary to acidify the samples to extract the product from the organic phase. The biotransformation mixtures were extracted by ethyl acetate and purified on RP-TLC plates (Figure S12, Supplementary Materials).

**Figure 5:**
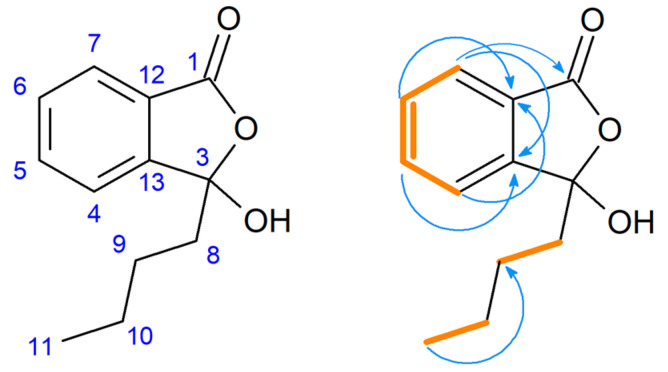
Pivotal Correlation Spectroscopy (orange bolded) and Nuclear Multiple Bond Coherence (arrows) correlations of 3-butyl-3-hydroxyphthalide (**2**).

Using NMR spectra, we confirmed that the main isolated product of the biotransformation was (-)-3-butyl-3-hydroxyphthalide (**2**). The assignment of aromatic signals of 3-butyl-3-hydroxyphthalide (**2**) in the range of δ = 7.56–7.81 ppm was mainly allowed by Correlation Spectroscopy (COSY) (coupling of H-5 with H-6 and H-4 protons). This was also facilitated via Nuclear Multiple Bond Coherence (HMBC) spectrum coupling of C-13 with protons H-5 and H-7 without correlation with H-4, as well as a coupling of C-12 with H-4 and H-6 without correlation with H-7 (Figure 5). The presence of a hydroxyl group was confirmed by the broad signal in the range of δ = 3.70–4.50 ppm (Figure S3, Supplementary Materials). The characteristic signal for the proton H-8 in the substrate structure at δ = 5.64 was absent in the spectra of this derivative. The signal for the carbon atom with the hydroxyl group (C-3) in the 3-butyl-3-hydroxyphthalide (**2**) spectrum was shifted upfield (δ = 107.9) when compared to that of the unsubstituted carbon atom (δ = 145.9) in the precursor compound (**1**) (Figure S4, Supplementary Materials).

Overall, the yield of the product (**2**) in the biotransformation mixture at the end of the process was in the range 57–82% depending on the strain used, as determined by HPLC. The yield after isolation was approximately 29–45% (Table 1). The specific rotations [α]$\begin{array}{c} 25\\ 589 \end{array}$ of product (**2**) isolated from particular strains were as follows: −2.4 (c = 1.0, CHCl_3_) for *A. cylindrospora* AM 336; −0.5 (c = 1.0, CHCl_3_) for *A. candidus* AM 386; and −3.6 (c = 1.0, CHCl_3_) for *C. indicum* AM 158.

### 2.2. Proposed Pathways for 3-Butyl-3-hydroxyphthalide (**2**) Formation

**Figure 6:**
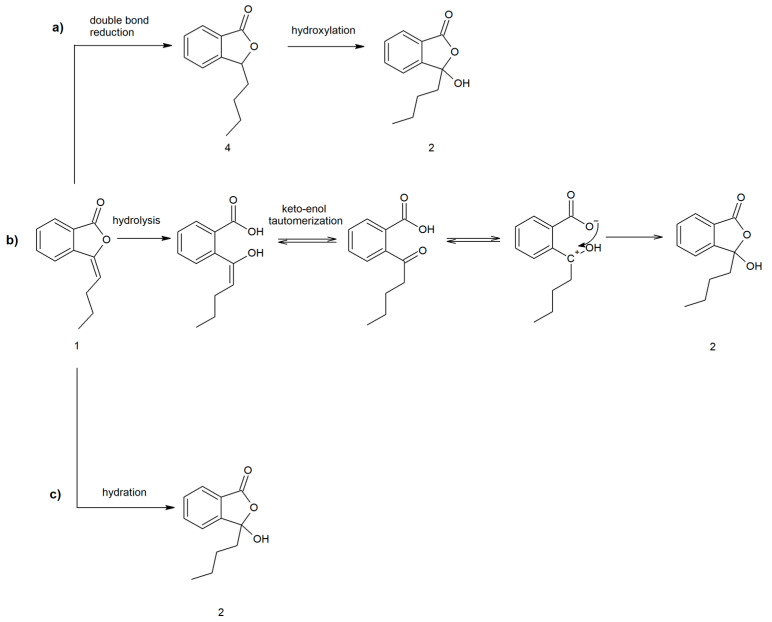
Proposed mechanism of 3-butyl-3-hydroxyphthalide (**2**) formation via (**a**) the intermediary product 3-*n*-butylphthalide (**4**); (**b**) hydrolysis of 3-*n*-butylidenephthalide (**1**) and (**c**) hydration of 3-*n*-butylidenephthalide (**1**).

Hydroxylation frequently occurs via cytochrome P450 enzymes involved in xenobiotic metabolism [35]. To examine the possibility of 3-butyl-3-hydroxyphthalide (**2**) formation through hydroxylation of the saturated analog (**4**) of 3-*n*-butylidenephthalide (**1**) (Figure 6), we also performed biotransformations of 3-*n*-butylphthalide (**4**) using the three previously used microorganisms.

However, our experiments showed that 3-*n*-butylphthalide (**4**) was not converted to product (**2**). We used ^13^C NMR as a diagnostic tool to analyze the biotransformation mixture. Spectra of the biotransformation mixtures showed a lack of the characteristic signal for the C-3 carbon in 3-butyl-3-hydroxyphthalide (**2**) at *δ =* 107.9 ppm (Figure S13, Supplementary Materials). Additionally, the saturated analog (**4**) of 3-*n*-butylidenephthalide (**1**) was not observed during the biotransformations. Therefore, the possibility of the conversion of the compound (**1**) through the reduction of a double bond followed by hydroxylation was rejected.

Another proposed mechanism involves the microbial hydrolysis of 3-*n*-butylidenephthalide (**1**), keto-enol tautomerism and the closing of the lactone moiety (Figure 6). Aldol-lactonizations may be selectively conducted by enzymes [36]; however, in the proposed mechanism, the molecule with a positive charge on C-3 carbon is formed (sp^2^ hybridization). Such flat carbocation is attacked by all sides of the plane, and thus, obtainment of a racemate is probable. Therefore, as we obtained an optically active product, we excluded this mechanism as a possibility.

The most probable pathway included hydration, which is the addition of water to a double bond in the C-3 position (Figure 6). The hydration route was also proposed by Yan et al. when they investigated the conversion pathway of ligustilide in rat plasma, as well as in later research concerning the metabolism of 3-*n*-butylidenephthalide (**1**). The researchers proposed the aromatization of ligustilide to 3-*n*-butylidenephthalide (**1**), with a further addition of water to obtain 3-butyl-3-hydroxyphthalide (**2**) [28,37]. Hydratases reportedly have certain advantages over synthetic catalyst-mediated enantioselective hydration, as the latter often requires harsh environmental conditions [38].

Unfortunately, to the best of our knowledge, there is no available data on phthalide hydratases. In order to exclude acid-catalyzed hydration as a possible mechanism, compound (**1**) was additionally dissolved in a water and organic solvent mixture (35:65) in the presence of HCl (pH = 1). Compound (**2**) was not observed during HPLC analysis of the sample.

**Figure 7:**
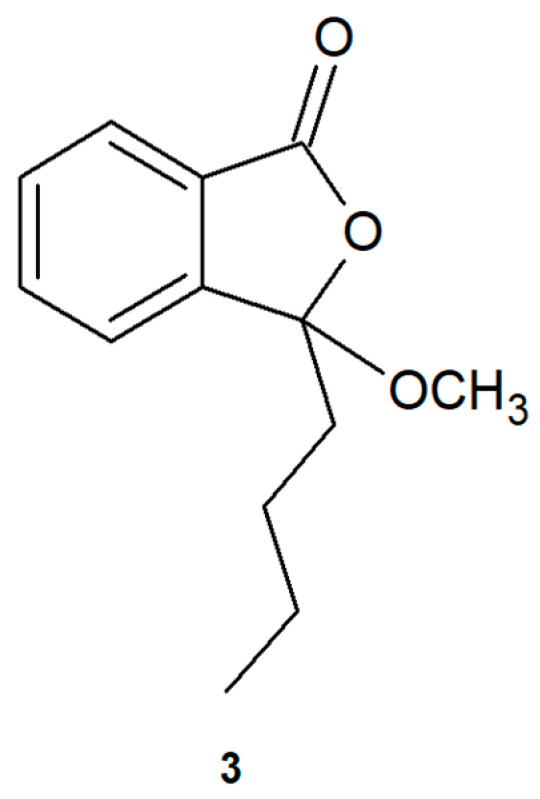
Product (3-butyl-3-methoxyphthalide) (**3**) of the esterification of the biotransformation mixture of *Aspergillus candidus* AM 386.

Interestingly, Li et al. observed the further methylation of product (**2**) in rat bile as a metabolite of a Chinese herbal formula containing phthalides, including 3-*n*-butylidenephthalide (**1**) [39]. Even though 3-butyl-3-methoxyphthalide (**3**) (Figure 7) was not observed during biotransformations in the cultures of fungal strains, we chemically synthesized this compound. We conducted hydrolysis and a reaction with boron trifluoride of the biotransformation mixture from *A. candidus* AM 386. We then examined whether the incorporation of a methoxy group affects the bioactivity. The substitution of the hydroxyl group in 3-butyl-3-methoxyphthalide (**3**) was evidenced by the presence of a three-proton singlet at 3.04 ppm (H-3a) (Figure S8, Supplementary Materials).

### 2.3. Influence of Lipophilicity and Fungistatic Activity

Xenobiotics are usually converted to more polar metabolites so that they are easily excreted from the organism. The converted compounds may exhibit pharmacological activity or become inactive [21]. We planned to determine whether there is a dependent relationship between the polarity of the metabolite (**2**) of 3-*n*-butylidenepthalide (**1**) and its bioactivity. In order to achieve this, we evaluated the pharmacodynamic parameters and fungistatic properties of these compounds.

To assess the lipophilicity of phthalides and their metabolites, the chromatographic partition coefficient (log kw) and hydrophobicity index (φ_0_) values were determined using RP-HPLC based on retention parameters. The partition coefficient (log *P*) values were calculated using theoretical methods proposed at ALOGPS 2.1.

**Figure 8:**
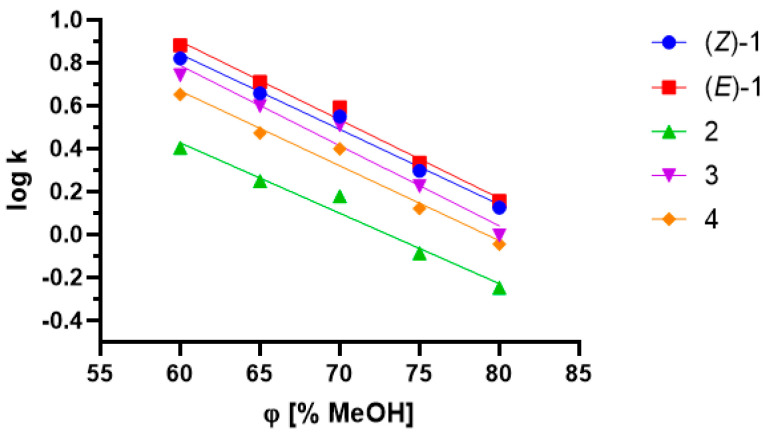
Log k and methanol volume fraction dependence for 3-*n*-butylidenephthalide (**1**); 3-butyl-3-hydroxyphthalide (**2**); 3-butyl-3-methoxyphthalide (**3**); and 3-*n*-butylphthalide (**4**).

RP-HPLC is a useful method for preliminary research into drug lipophilicity due to the small amount of compound required [40]. The method is based on the evaluation of the retention factor (k). It has been previously stated that there is a linear correlation between the log k value and the volume fraction of organic solvent [41]. This dependence, involving the log k values of five compounds, is presented in Figure 8.

The chromatographic lipophilicity parameters are presented in Table 2. Overall, high coefficient values of determination (≥0.9633) were obtained for all the examined compounds. The highest log k_w_ value was achieved for 3-*n*-butylidenephthalide (**1**) at −3.094 for isomer (*E*). The metabolite of the abovementioned compound, 3-butyl-3-hydroxyphthalide (**2**), had a notably higher polarity (log k_w_ = 2.397). This result confirmed the introduction of a new polar group into the compound, which is typical for phase 1 reactions [21]. Introduction of a methyl group into the structure of 3-butyl-3-methoxyphthalide (**3**) resulted in increased hydrophobicity (φ_0_ = 81.200) in comparison to (**2**) (φ_0_ = 73.079). The lack of a double bond in the side chain of 3-*n*-butylphthalide (**4**) resulted in a higher polarity (log k_w_ = 2.7623) compared with that of compound (**1**). The log *P* values according to ALOGPS 2.1 did not exceed 3.38 for the abovementioned compounds. 

It has been previously stated that 90% of compounds that reach phase 2 clinical trials do not exceed a log *P* value of 5 [33]. Studies conducted by Tamaian et al. (2015) on thiazolyl-carbonyl-thiosemicarbazides and thiazolyl-azoles emphasized the importance of optimum hydrophilic-lipophilic balance. These studies showed that the highest bioactivity was observed for the compounds that had medium values for the lipophilicity parameters [42]. Log *P* values that are too high may result in poorer drug solubility. For instance, the widely used fungistatic ketoconazole with a log *P* value of 3.73 reportedly belongs to class II of the Biopharmaceutics Classification System, suggesting that the compound has high permeability and low solubility [43,44]. Conversely, another antimycotic fluconazole has a log *P* value of 0.5 [45]. In theory, such low lipophilicity should be connected with lower membrane permeability [46].

Considering the bioactive potential of phthalides, including 3-*n*-butylidenephthalide (**1**) and 3-*n*-butylphthalide (**4**) [7,8,9,10,11,47,48], we determined the antimicrobial potential of these compounds. Moreover, we also tested whether their derivatives (3-butyl-3-hydroxyphthalide (**2**) and 3-butyl-3-methoxyphthalide (**3**)) show similar inhibitory properties against selected strains of *C. albicans*. We were particularly interested in whether metabolite (**2**), with the hydroxyl group in the C-3 position, and its methoxy analogue (**3**) would also inhibit microorganism growth and whether their lipophilicity influences their activity.

Phthalides from Apiaceae plants, such as 3-*n*-butyl-4,5-dihydrophthalide and sedanolide, have been previously tested against *Candida* yeasts. These phthalides were mostly found to be capable of completely inhibiting these yeasts at a concentration of 100 μg/mL [47,48]. 3-*n*-Butylphthalide (**4**) showed fungistatic properties against clinical isolates of *Candida albicans* with an MIC_80_ of 128 µg/mL [13]. However, 3-*n*-butylidenephthalide (**1**) has not been previously studied against this microorganism.

Overall, we observed that phthalides (**1**) and (**4**) were active against all tested *Candida* strains and had MIC_50_ values ranging from below the smallest tested concentration (23 µg/mL) to 123 µg/mL (Table 3). In two cases, the activity of unsaturated lactone (**1**) was higher than that of its analog, which did not have the double bond in its side chain (**4**).

3-*n*-Butylidenephthalide (**1**) efficiently inhibited the growth of the *C. albicans* clinical isolates 595/20 and 38, with an MIC_50_ below 50 µg/mL. It also inhibited strain 636/20, showing an MIC_50_ value of 88 µg/mL. By contrast, strain 636/20 was not inhibited by the fluconazole at the highest concentration used (250 µg/mL). This phthalide was slightly less active against *C. albicans* ATTC 90028 and had an MIC_50_ of 110 µg/mL. 3-*n*-Butylphthalide (**4**) was also significantly active against the clinical isolate *C. albicans* 595/20, showing an MIC_50_ below 50 µg/mL. These inhibitory activities of phthalides (**1**) and (**4**) might correlate with their high lipophilicity, and thereby with their sufficient membrane permeability.

It appeared that (-)-3-butyl-3-hydroxyphthalide (**2**) was slightly active only in the case of strains 636/20 and 38, with MIC_50_ values of 203 and 250 µg/mL, respectively. The compound did not influence the growth of other strains. The lack of activity of 3-butyl-3-hydroxyphthalide (**2**) suggests that 3-*n*-butylidenephthalide (**1**) is subjected to an inactivation pathway during its conversion. The lack of activity of (**2**) corresponds with significantly lower logk_w_ values, which may indicate that the permeability of the compound is too low. Introduction of the methoxy group in the C-3 position resulted in enhanced activity toward strain 595/20 (MIC_50_ = 115 µg/mL) compared with that of the metabolite, but it did not improve its fungistatic activity toward strain 636/20 (MIC_50_ = 244 µg/mL). However, the lipophilicity of (**2**) had a value close to that displayed by 3-*n*-butylidenephthalide (**1**). These results suggest that lipophilicity is not the main determinant of the antifungal activity of phthalides. In the case of 3-butyl-3-methoxyphthalide (**3**), its molecular structure and the occurrence of spatial hindrance may also influence its decreased activity.

Structure-activity dependences for phthalides have been previously proposed. Considering the dependence between the structure and fungistatic activity, available data focuses on preliminary structure-activity tests of more complex 3-substituted phthalides against selected phytopathogens. It has been stated that -OH and -NH_2_ groups incorporated in the benzene ring may increase the antifungal activity of phthalides due to the formation of a hydrogen bond with the active site of the pathogen enzyme [49]. In fact, the lack of the activity of the tested compounds (**2**) and (**3**) may be explained by the –OH and –OCH_3_ groups at the C-3 position. Xiao et al. showed the linkage between the structure of marine fungal phthalide derivatives and peroxisome proliferator-activated receptor gamma (PPAR-γ) binding and activation properties. This study also showed that the occurrence of an –OH group in the benzene ring has a positive effect on higher bioactivity; conversely, the presence of a double side chain at C-3 in phthalides resulted in lower binding and activation properties compared to compounds with a single chain [50].

## 3. Materials and Methods

### 3.1. Compounds

The substrate for the biotransformations—3-*n*-butylidenephthalide (**1**) was purchased from Sigma-Aldrich (St. Louis, MO, USA) as the mixture of (*E*) and (*Z*) isomers, at the ratio of 9:1 according to the ^1^H NMR and HPLC (wavelength 274 nm).

3-butyl-3-methoxyphthalide (**3**) was obtained through 24 h alkaline hydrolysis of a mixture of products (80 mg) obtained in the biotransformation catalyzed by *A. candidus* AM 386. The biotransformation was conducted using 3 mL 0.5M KOH in MeOH, and esterification was carried out using 4 mL 20% BF_3_ in methanol. After 1 h, the mixture was briefly filtered using silica gel to remove BF_3_. The mixture was then eluted by diethyl ether_._ Crude product was concentrated in a rotary evaporator and purified using silica gel and a hexane:ethyl acetate 19:1 (*v*/*v*) eluent. The yield of 3-butyl-3-methoxyphthalide (**3**) was 41.52 mg (51.9%). 

3-*n-*Butylphthalide (**4**) was synthesized as follows: *n*-butyllithium in hexane (*n*-BuLi; Sigma-Aldrich, St. Louis, MO, USA) was added dropwise to the phthalic anhydride dissolved in tetrahydrofuran (7.4 g, 0.05 mol) at −78 °C (isopropanol/CO_2_ (s)). The molar ratio of the anhydride to *n*-BuLi was 3:1. The reaction was run for 20 min and then quenched with 10% hydrochloric acid, extracted with diethyl ether, washed with water and dried with MgSO_4_. The solvent was then evaporated. The crude extract was dissolved in 30 mL THF and NaBH_4_ (1.13 g, 0.03 mol; Sigma-Aldrich, St. Louis, MO, USA) was added. The reaction was stopped by adding 10% hydrochloric acid. THF was evaporated and the mixture was extracted with diethyl ether and washed with water. The organic layer was dried with MgSO_4_ and collected. After concentration, the crude product was purified by column chromatography using petroleum ether:acetone 3:1 (*v*/*v*) as eluent. The yield of 3-*n-*butylphthalide (**4**) was 2.12 g (67%). The progress of the reactions was monitored by analytical TLC and GC. The structures of the compounds (**3**,**4**) were confirmed by NMR spectroscopy.

### 3.2. Microorganisms

The following strains were used in the biotransformations: *Mucor spinosus* AM 398, *Absidia cylindrospora* AM 336, *Pycnidiella resinae* AM 50, *Penicillium chrysogeum* AM 112, *Chaetomium indicum* AM 158, *Fusarium culmorum* AM 9, *Aspergillus candidus* AM 386, *Laetisporus sulphurens* AM 515, *Fusicoccum amygdali* AM 258 and *Ascosphaera apis* AM 496. These were all obtained from the Department of Chemistry collection at the Wrocław University of Environmental and Life Sciences. These strains were maintained on Sabouraud or Czapek agar slants at 4 °C.

Fungistatic activity was detected against *Candida albicans* ATCC 90028 from American Type Culture Collection, and its clinical isolates *Candida albicans* 636/20, *Candida albicans* 595/20 and *Candida albicans* 38 were obtained from Wrocław Medical University, Poland.

### 3.3. Biotransformations

The biotransformations were conducted in 300 mL Erlenmeyer flasks. Each strain was inoculated using the sterile Sabouraud medium (75 mL), which consisted of 30 g glucose (Chempur, Piekary Śląskie, Poland), 10 g bactopeptone (Biocorp, Warszawa, Poland) and 1 L distilled water (pH 6.88). After 5 days of incubation at 25 °C on a rotary shaker, 3-*n*-butylidenephthalide (**1**) was added (20 mg dissolved in 0.5 mL of acetone) and samples were collected every 2 days to measure pH values. Product yield was determined on days 2, 4, 6, 8 and 14. The samples were acidified to pH 5 using hydrochloric acid (if necessary) and extracted using ethyl acetate. The samples were then dried using MgSO_4_ and subjected to evaporation at 25 °C. Next, the samples were dissolved in methanol, filtered using a 0.45 µm PTFE filter and analyzed by HPLC.

Three microorganisms were chosen for the scale-up process: *A. cylindrospora* AM 336, *C. indicum* AM 158 and *A. candidus* AM 386. The preparative biotransformations were conducted in 2000 mL Erlenmeyer flasks using 500 mL of a sterile Sabouraud medium and precultivated inoculum, which constituted 10% of the medium volume. After 5 days of incubation at 25 °C on a rotary shaker, 3-*n*-butylidenephthalide (**1**) was added (150 mg dissolved in 1 mL acetone) to the culture. The samples were collected every 2 days and the pH values were measured. The samples were prepared as previously described (except for the evaporation step) and subjected to GC analysis in ethyl acetate. Product mixtures were collected on day 8 of the process for *A. candidus* AM 386 and on day 14 for *C. indicum* AM 158 and *A. cylindrospora* AM 336. The collected samples were acidified with 10% hydrochloric acid, extracted twice using ethyl acetate and analyzed by RP-TLC.

The product was purified using 250 µm thick TLC Silica gel 60 RP-18 F254s plates (Merck, Darmstadt, Germany) with 70% MeCN and 30% H_2_O acidified by 1% HCOOH eluent. After separation, the plates were visualized using a UV lamp at 254 and 365 nm. The product was scraped off and extracted with ethyl acetate, after which the organic layer was collected, dried with MgSO_4_ and evaporated by the rotary evaporator. The structures of the compounds were determined by NMR. 

### 3.4. Analysis

TLC analysis was performed on glass plates covered with silica gel 60 F254 (Merck) and developed using hexane:acetone 2:1 (*v*/*v*) eluent. After elucidation, the plates were visualized by a solution of 1% Ce(SO_4_)_2_ and 2% phosphoromolybdic acid in 10% H_2_SO_4_.

HPLC analysis was performed using a Dionex UltiMate 3000 instrument equipped with a diode array detector (Thermo Fisher Scientfic, Waltham, MA, USA) with a Phenomenex Luna^®^ 5 µm C18 100 Å, 250 × 4.6 mm column (Phenomenex, Torrance, CA, USA). The mobile phase was composed of water acidified with 5% formic acid (A) and methanol (B). Gradient elution conditions were as follows: 0–10 min, 35% A/65% B; 10–20 min, 30% A/70% B; 21–30 min, 0% A, 100% B; 31–35 min, 65% A/35% B; and 35–45 min, 35% A, 65% B. To observe the product, the following parameters were selected: flow rate, 1.0 mL/min; injection volume, 8 μL; column incubation temperature, 30 °C; and detection wavelength, 274 nm.

GC analysis (FID, H_2_ as carrier gas) was performed on an Agilent Technologies 7890 N GC System (Santa Clara, CA, USA) using a Cyclosil-B column (30 m × 0.25 mm × 0.25 μm, Agilent Technologies). The temperature program was 120 °C, 200 °C (8 °C/min), and 240 °C (10 °C/min).

Molecular weights of compounds were assessed using GC-MS on a Saturn 2000 MS Varian Chrompack CP-3800 (Walnut Creek, CA, USA). 

The optical rotation of 3-butyl-3-hydroxyphthalide (**2**) was measured using JASCO P-2000-Na digital polarimeter (ABL & E-JASCO, Kraków, Poland) in chloroform. The specific rotations [α]$\begin{array}{c} 25\\ 589 \end{array}$ of product (**2**) isolated from particular strains were as follows: −2.4 (c = 1.0, CHCl_3_) for *A. cylindrospora* AM 336; −0.5 (c = 1.0, CHCl_3_) for *A. candidus* AM 386; and −3.6 (c = 1.0, CHCl_3_) for *C. indicum* AM 158.

NMR spectra (^1^H NMR, ^13^C NMR) of compounds (**1**), (**3,4**) were recorded on a Bruker Avance DRX-500 spectrometer (Bruker, Billerica, MA, USA) in CDCl_3_. 3-butyl-3-hydroxyphthalide (**2**) (^1^H NMR, ^13^C NMR, COSY, HSQC, HMBC) was measured on JNM-ECZS 400 MHz NMR spectrometer (JEOL USA, Peabody, MA, USA). The spectral data are presented below as well as in the attached Supplementary Materials.

3-*n*-butylidenephthalide (**1**).

^1^H NMR (500 MHz), δ (ppm): 0.98 (t, 3H, *J* = 7.40, H-11), 1.54 (m, 2H, H-10), 2.45 (q, 2H, *J_1_* = 15.00, *J_2_* = 7.50, H-9), 5.63 (t, 1H, *J* = 7.80, H-8), 7.49 (t, 1H, *J* = 7.30, H-5), 7.64 (m, 2H, H-4), 7.66 (m, 2H, H-6), 7.88 (d, 1H, *J* = 7.70, H-7).

^13^C NMR (151 MHz), δ (ppm): 13.9 (C-11), 22.6 (C-10), 28.0 (C-9), 109.6 (C-8), 119.8 (C-4), 124.6 (C-12), 125.4 (C-7), 129.5 (C-6), 134.3 (C-5), 139.7 (C-3), 145.9 (C-13), 167.3 (C-1).

GC-EIMS 189 (M + 1).

3-butyl-3-hydroxyphthalide (**2**).

^1^H NMR: (400 MHz), δ (ppm): 0.85 (t, 3H, H-11, *J* = 7.22 Hz), 1.14 (m, 1H, one of H-9), 1.31 (m, 2H, H-10), 1.38 (m, 1H, one of H-9), 2.07 (ddd, 1H, one of H-8, *J*_1_ = 14.0, *J*_2_ *=* 11.8, *J*_3_ = 4.5 Hz), 2.20 (m, 1H, one of H-8) 7.56 (m, 2H, H-4, H-6), 7.70 (t, 1H, H-5, *J* = 7.48 Hz), 7.81 (d, 1H, H-7, *J* = 7.52 Hz).

^13^C NMR (101 MHz), δ (ppm): 13.9 (C-11), 22.6 (C-10), 25.5 (C-9), 38.7 (C-8), 107.9 (C-3), 122.4 (C-4), 125.6 (C-7), 126.9 (C-12), 130.7 (C-6), 134.8 (C-5), 149.0 (C-13), 168.9 (C-1).

3-butyl-3-hydroxyphthalide (2) due to the low volatility was assessed at the GC-EIMS after esterification as 3-butyl-3-methoxyphthalide (3)-GC-EIMS 221 (M + 1).

3-butyl-3-methoxyphthalide (**3**).

^1^H NMR: (500 MHz), δ (ppm): 0.83 (t, 3H, H-11, *J* = 7.3), 1.12 (m, 1H, one of H-10), 1.28 (m, 2H, H-9), 1.39 (m, 1H, one of H-10), 2.01 (ddd, 1H, one of H-8, *J*_1_ = 4.58, *J*_2_ = 11.74, *J*_3_ = 14.05), 2.16 (ddd, 1H, one of H-8, *J*_1_ = 4.72, *J*_2_ = 11.85, *J*_3_ = 14.04) 3.04 (s, 3H, H-3a), 7.47 (d, 1H, H-4, *J* = 7.6), 7.59 (td, 1H, H-6, *J*_1_ = 0.82, *J*_2_ = 7.55, *J*_3_ = 7.53), 7.71 (td, 1H, H-5, *J*_1_ = 0.99, *J*_2_ = 7.50, *J*_3_ = 7.50), 7.88 (d, 1H, H-7, *J* = 7.65).

^13^C NMR (151 MHz), δ (ppm): 13.9 (C-11), 22.7 (C-10), 25.3 (C-9), 38.3 (C-8), 51.2 (C-3a), 111.1 (C-3), 122.6 (C-4), 125.6 (C-7), 128.2 (C-12), 130.7 (C-6), 134.6 (C-5), 146.8 (C-13), 168.5 (C-1).

GC-EIMS 221 (M + 1).

3-*n*-butylphthalide (**4**).

^1^H NMR (500 MHz), δ (ppm): 0.91 (t, 3H, *J* = 7.2, H-11), 1.39 (m, 2H, H-10), 1.48 (m, 2H, H-9), 1.76 (m, 1H, one of H-8), 2.04 (m, 1H, one of H-8), 5.47 (dd, 1H, *J*_1_ = 7.9, *J*_2_ = 3.7, H-3), 7.44 (d, 1H, *J* = 7.7, CH-4), 7.52 (t, 1H, *J* = 7.5, H-6), 7.66 (t, 1H, *J* = 7.5, H-5), 7.89 (d, 1H, *J* = 7.7, H-7).

^13^C NMR (151 MHz), δ (ppm): 14.0 (C-11), 22.6 (C-10), 27.0 (C-9), 34.6 (C-8), 81.6 (C-3), 121.8 (C-4), 125.8 (C-7), 126.3 (C-12) 129.1 (C-6), 134.1 (C-5), 150.3 (C–13), 170.8 (C-1).

GC-EIMS 191 (M + 1).

### 3.5. Lipophilicity

The concentration of tested compounds (**1**–**4**) was 0.1 mg/mL. Dead time (t_0_) was measured by injecting 1% aqueous NaNO_3_ solution_._ The injection volume was 10 μL, the temperature was 35 °C and the flow rate was 1.5 mL/min. The retention time (t_R_) of the samples was also assessed by RP-HPLC in triplicate using the abovementioned column and isocratic elution. The eluents consisted of methanol and water, both of which were acidified by 1% formic acid *v*/*v* at 60–80% of the organic phase. The retention factor (k) was calculated according to the following equation: *k =* (t_R_ − t_0_)/t_0._ A graph of log k as a function of the volume fraction of organic solvent was plotted. The value of the chromatographic lipophilicity index, log k_w_ (log k value with 0% methanol in the mobile phase), was determined by extrapolating this correlation. The chromatographic hydrophobicity index φ_0_ was also assessed as the volume fraction of methanol that yields a log k value of 0 [31]. Theoretical log *P* (partition coefficient**)** values were calculated with the use of ALOGPS 2.1. Pearson’s correlation coefficients between the log *P* and log k_w_ were determined using GraphPad Prism, and differences with *p* < 0.05 were considered statistically significant.

### 3.6. Fungistatic Activity

Compounds (**1**–**4**) were tested against four *Candida albicans* strains (*C. albicans* ATTC 90028 and its clinical isolates *C. albicans* 636/20, *C. albicans* 595/20 and *C. albicans* 38) using the broth microdilution technique. The medium used for the tests was YPD, consisting of 20 g glucose (Chempur, Piekary Śląskie, Poland), 20 g bactopeptone (Biocorp, Warszawa, Poland), 10 g yeast extract (BTL, Łódź, Poland) and 1 L distilled water; the pH was adjusted to 6.5. The solutions of the compounds were prepared in dimethyl sulfoxide (DMSO) and diluted in YPD to obtain final concentrations in the range of 50–250 µg/mL. Fluconazole was tested at the range of 0.064–64 µg/mL with the exception of the resistant strain 636/20, which was additionally tested in the range of 50–250 µg/mL. Next, 100 µL of each solution was pipetted into wells of a 96-well microtiter plate. The inoculum was standardized to 0.5 McFarland standard and then diluted to obtain the final suspension with a cell density of 0.5 × 10^3^ to 2.5 × 10^3^ CFU/mL. The inoculum size used was 100 µL. The positive control comprised DMSO added in the same concentration as the tested compounds in the inoculum, while the negative control was DMSO diluted in the broth without the addition of inoculum. All samples were tested at least in triplicate. The microtiter plates were incubated at 35 °C for 24 h in a Biosan PST-60 HL ThermoShaker (Riga, Latvia) at 1000 rpm. The fungistatic activity of the compounds was assessed by measuring the absorbance at a wavelength of 595 nm (Epoch, BioTek, Winooski, VT, USA) to determine the MIC_50_ value (i.e., the concentration of a compound required to inhibit the growth of 50% of microorganisms).

## 4. Conclusions

In this study, we confirmed the utility of microbially catalyzed biotransformation for obtaining the mammalian metabolite (**2**) of bioactive 3-*n*-butylidenephthalide (**1**). We show that microbiological transformations can efficiently produce the target compound and can be used to better understand phthalide lactone metabolism. Overall, 10 fungal strains were tested, five of which efficiently produced hydroxy-3-butylphthalide (**2**). We proposed three mechanisms by which 3-*n*-butylidenephthalide (**1**) conversion may occur. Even though hydroxylation remains popular in xenobiotic metabolism, we excluded this pathway based on ^13^C NMR spectra and suggested the hydration pathway as the most probable mechanism. Valuable contributions of this research include the analysis of the lipophilicity and fungistatic activity of both phthalides (**1**) and (**4**) and the metabolite (**2**). Antifungal assays revealed the potential of both 3-*n*-butylidenephthalide (**1**) and 3-*n*-butylphthalide (**4**) against clinical isolates of *C. albicans*, with an MIC_50_ value below 50 µg/mL. We did not observe the influence of a double bond-containing side chain on the activity of compounds (**1**) and (**4**). We noticed weak or even a lack of inhibitory properties for metabolite (**2**), which has lower lipophilicity and presumably lower permeability than compounds (**1**) and (**4**). We did not observe a straightforward correlation between lipophilicity and fungistatic activity. Apart from the lipophilicity, the spatial structure of compounds is the factor that influences their biological activity. The presence of –OH groups in compound (**2**) or –OCH_3_ groups in compound (**3**) causes the decrease of antifungal activity compared to that of 3-*n*-butylphthalide (**4**). Compound (**1**), which has a double bond in its structure, shows similar polarity to 3-butyl-3-methoxyphthalide (**3**); however, this compound had high biological activity. Considering the possible applications for phthalides, there is a necessity for further research, particularly regarding the biological activity of their metabolites.

## Figures and Tables

**Table 1:** Product (**2**) content in the biotransformation mixtures at the end of the processes both before and after isolation.

Strain	Time of the Process [Days]	3-Butyl-3-hydroxyphthalide (2)	
		(Based on HPLC) [%]	(Isolation Yield) [%]
*Chaetomium indicum* AM 158	14	69.8	36.6
*Absidia cylindospora* AM 336	14	57.0	29.2
*Aspergillus candidus* AM 386	8	82.2	45.4

**Table 2:** Chromatographic partition coefficient (log kw) and hydrophobicity index (φ_0_) for substrate (**1**) and products of its conversion (3-butyl-3-hydroxyphthalide (**2**), 3-butyl-3-methoxyphthalide (**3**) and 3-*n*-butylphthalide (**4**)) along with the calculated partition coefficient.

Compound	Log k_w_ **	Standard Error for the Slope	Standard Error for the Intercept	Coefficient of Determination	φ_0_ **	Log *P* (Calculated)
*(Z)*-1	2.9422	0.0025	0.1747	0.9851	84.063	3.38
*(E)-1 *	3.094	0.0024	0.1673	0.9875	84.536	3.38
2	2.397	0.0032	0.2256	0.9721	73.079	2.09
3	3.029	0.0042	0.2958	0.9633	81.200	2.86
4	2.7623	0.0032	0.2256	0.9752	79.149	3.00

* The separation of (*Z*) and (*E*) isomers are shown on the chromatogram (Figure S14, Supplementary Materials); ** The r value, indicating the correlation between measured log k_w_ and calculated log *P* values, is 0.8675.

**Table 3:** Minimal inhibitory concentration (MIC_50_) [µg/mL] for substrate (**1**), the product of its conversion: 3-butyl-3-hydroxyphthalide (**2**), its derivatives 3-butyl-3-methoxyphthalide (**3**) and 3-*n*-butylphthalide (**4**) when compared with that of fluconazole.

Compound	*C. albicans* 636/20	*C. albicans* 595/20	*C. albicans* 38	*C. albicans* ATTC 90028
**1**	88	<50	<50	110
**2**	203	>250 ^1^	250	>250 ^1^
**3**	244	115	>250 ^1^	>250 ^1^
**4**	123	<50	87	89
Fluconazole	>250 ^1^	0.89	0.44	4.50

^1^ Compounds inactive at the highest tested concentration (250 µg/mL).

## Data Availability

The data presented in this study are available on request from the corresponding author.

## Supplementary Materials

The following are available online at https://www.mdpi.com/article/10.3390/ijms22147600/s1.
