# Supplementary material for: Microbial Synthesis and Evaluation of Fungistatic Activity of 3-Butyl-3-hydroxyphthalide, the Mammalian Metabolite of 3-n-Butylidenephthalide
Source: Int J Mol Sci. 2021 Jul 15;22(14):7600. doi: 10.3390/ijms22147600 (PMC8304955; doi:10.3390/ijms22147600)
Supplement: Supplementary file 1 [file ijms-22-07600-s001.zip › ijms-1250316-supplementary.pdf]

Supplementary Materials

Microbial Synthesis and Evaluation of Fungistatic Activity of 3-Butyl-3-hydroxyphthalide, the Mammalian Metabolite of 3-*n*-Butylidenephthalide

Joanna Gach *, Teresa Olejniczak *, Piotr Krężel and Filip Boratyński

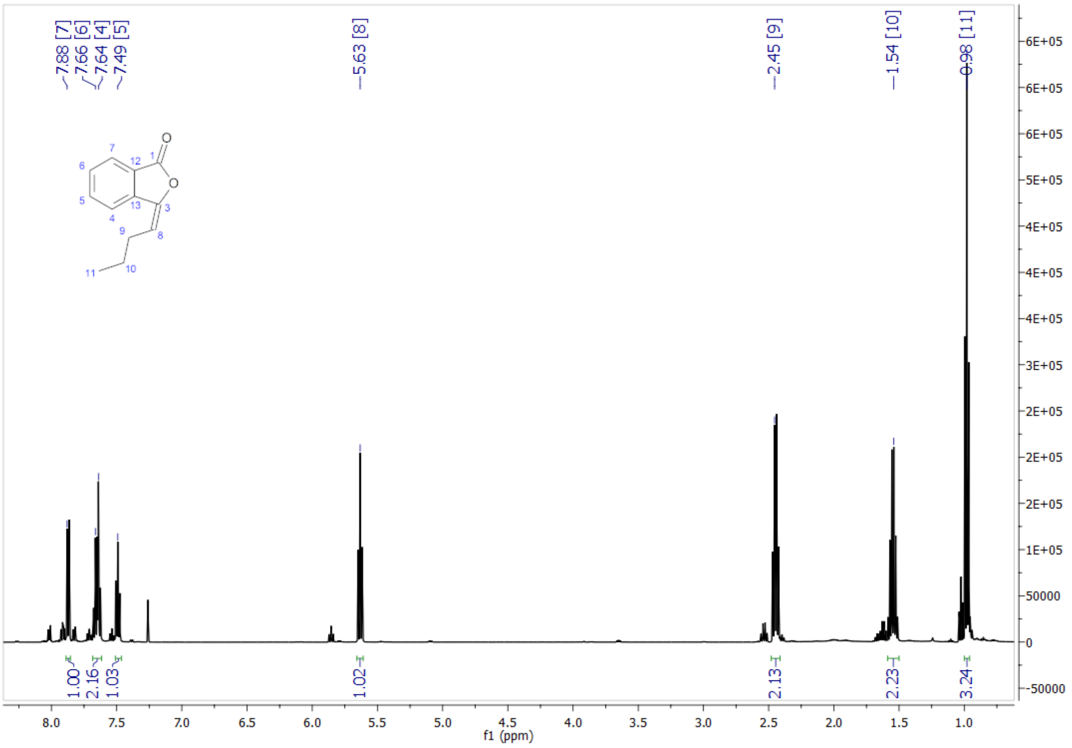

Figure S1. ^{1}H NMR spectrum of 3-*n*-butylidenephthalide (**1**).

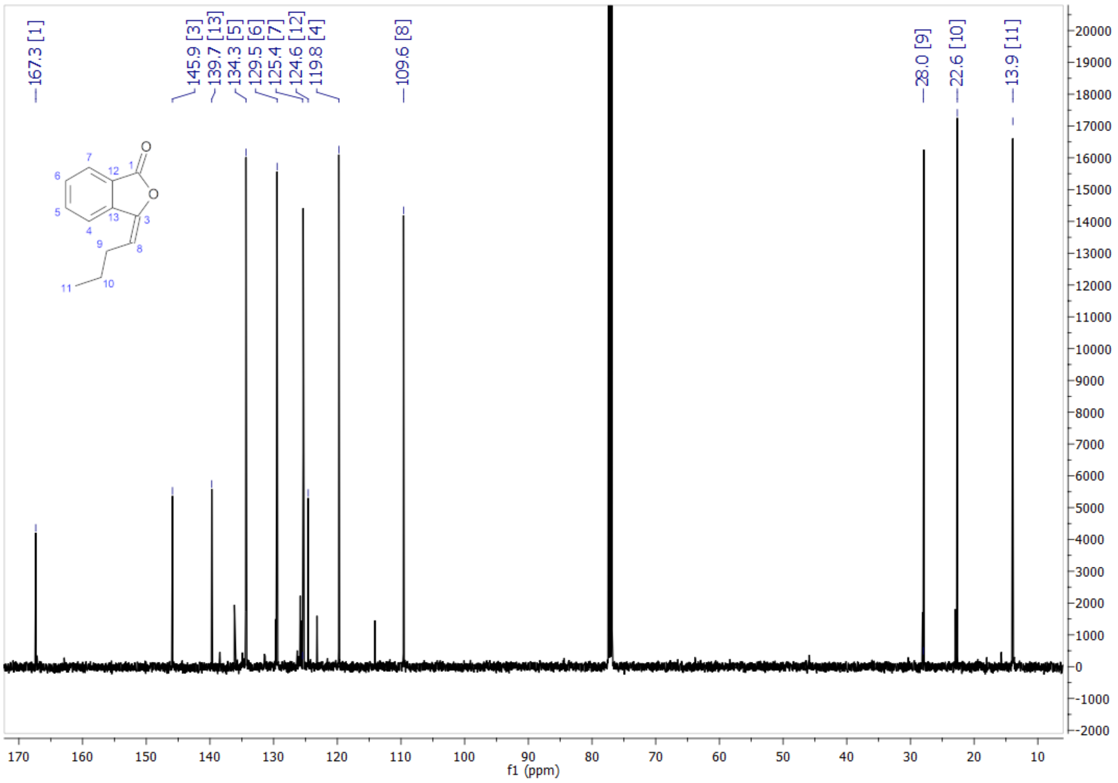

Figure S2. ^{13}C NMR spectrum of 3-*n*-butylidenephthalide (**1**).

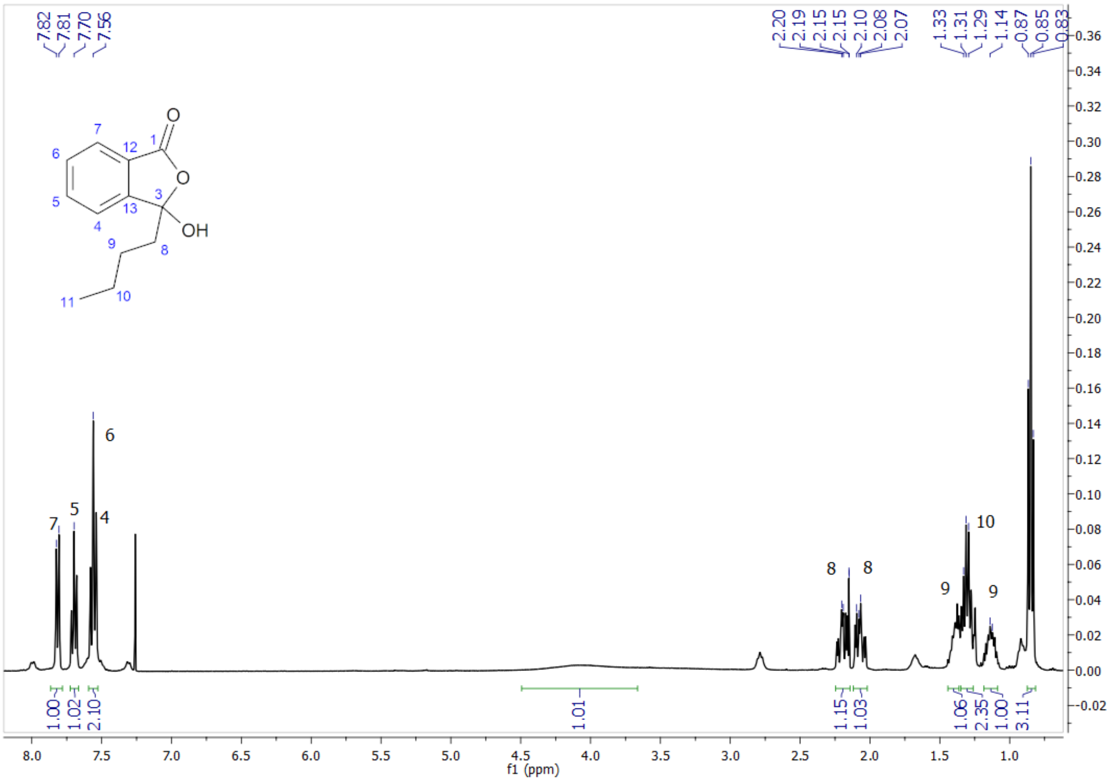

Figure S3. ^{1}H NMR spectrum of 3-butyl-3-hydroxyphthalide (**2**).

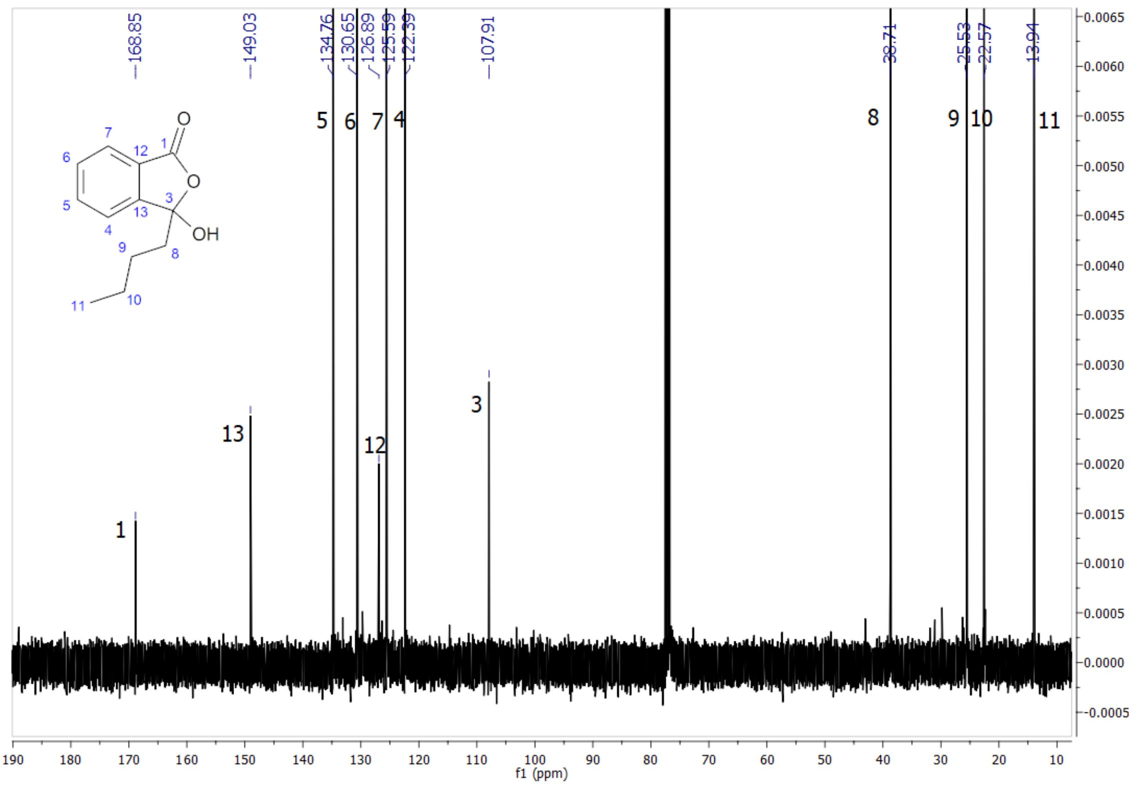

Figure S4. ^{13}C NMR spectrum of 3-butyl-3-hydroxyphthalide (**2**).

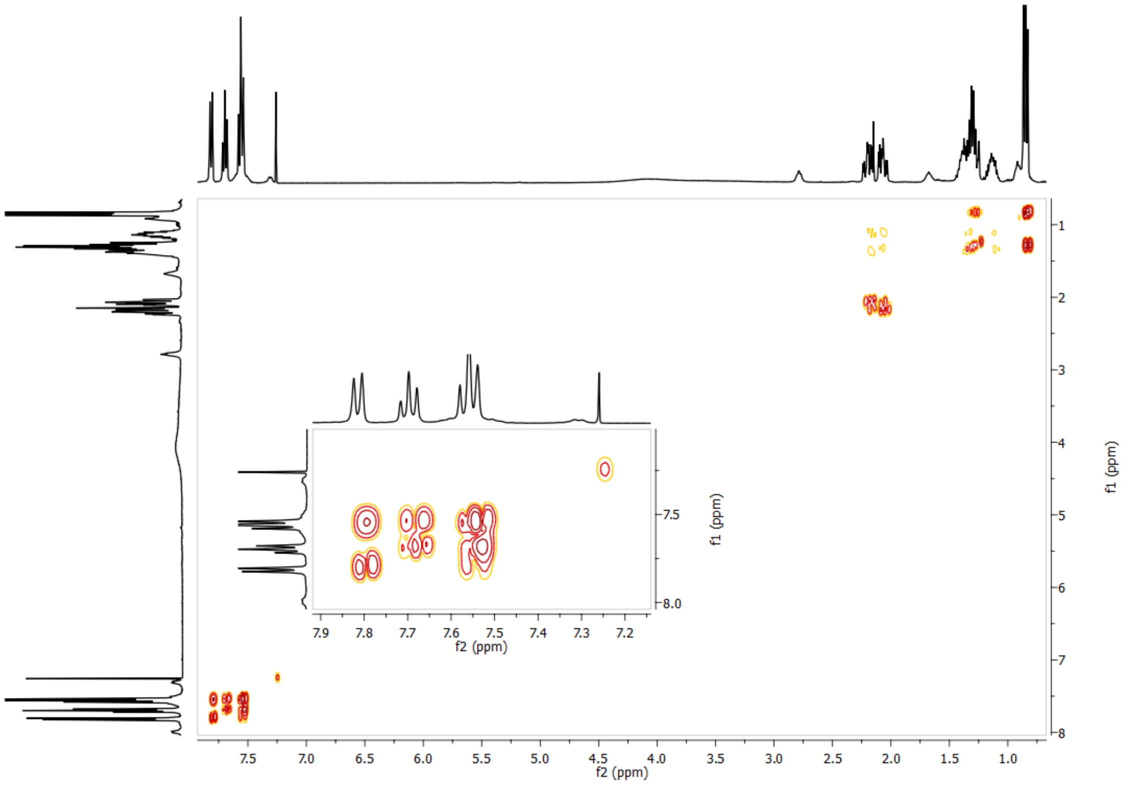

Figure S5. COSY spectrum of 3-butyl-3-hydroxyphthalide (**2**).

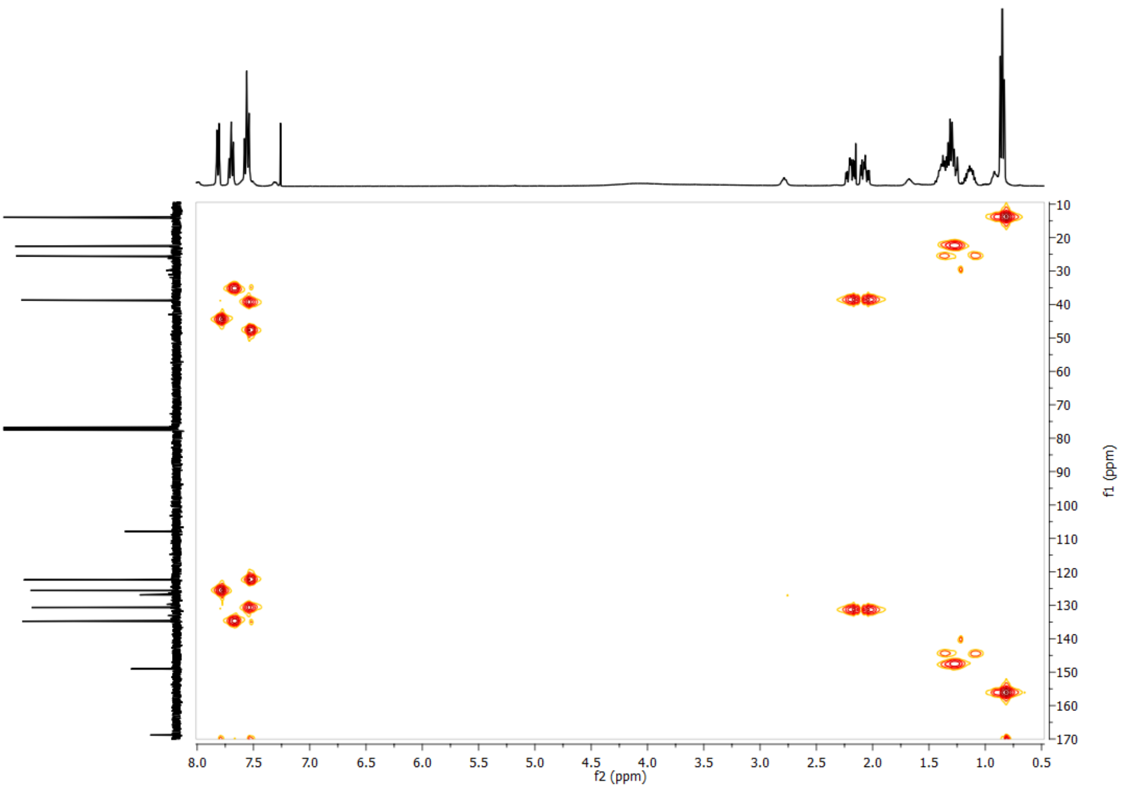

Figure S6. HSQC spectrum of 3-butyl-3-hydroxyphthalide (**2**).

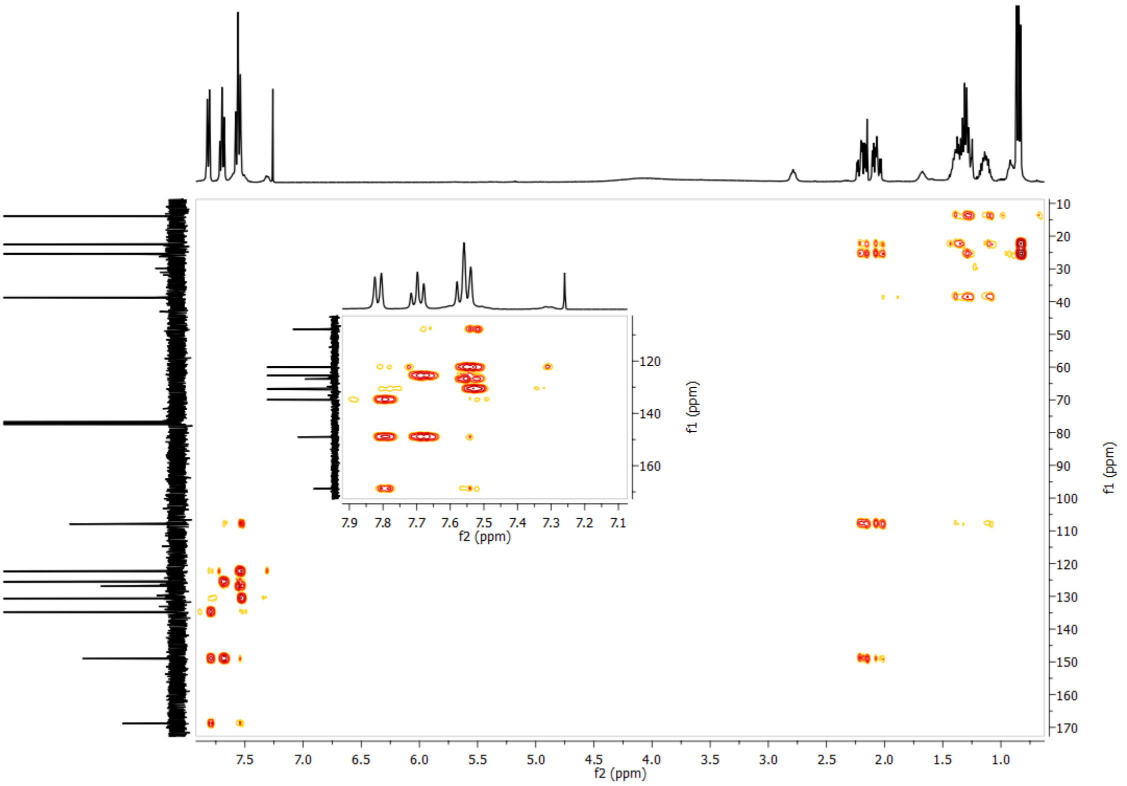

Figure S7. HMBC spectrum of 3-butyl-3-hydroxyphthalide (**2**).

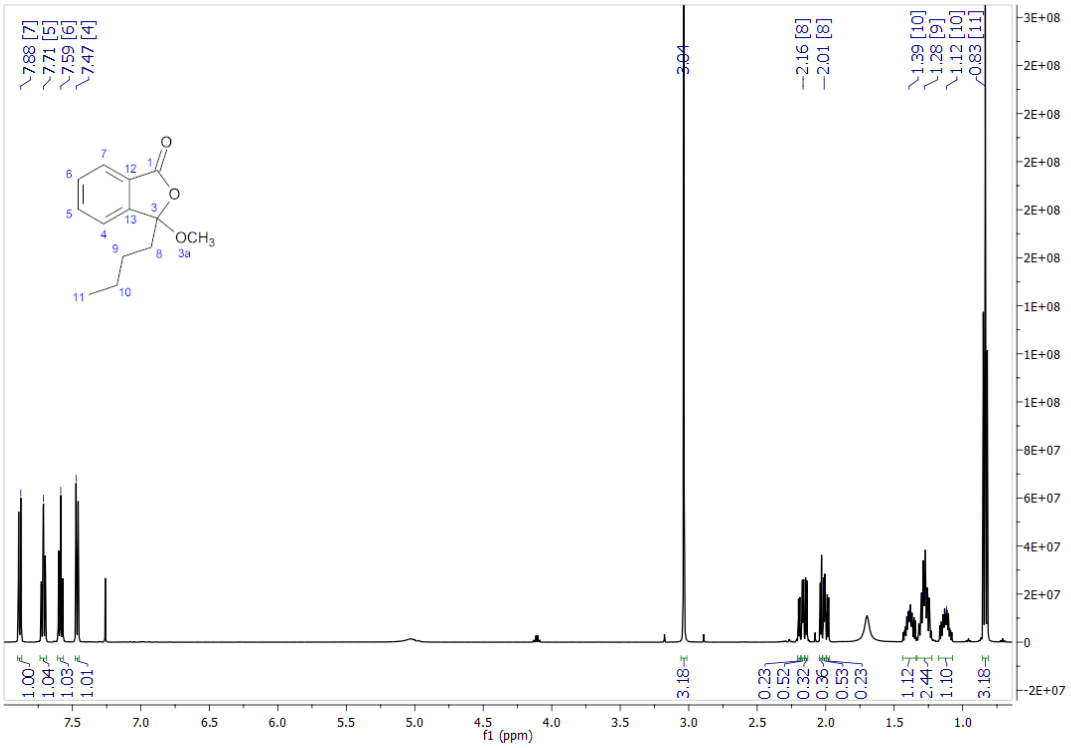

Figure S8. ^{1}H NMR spectrum of 3-butyl-3-methoxyphthalide (**3**).

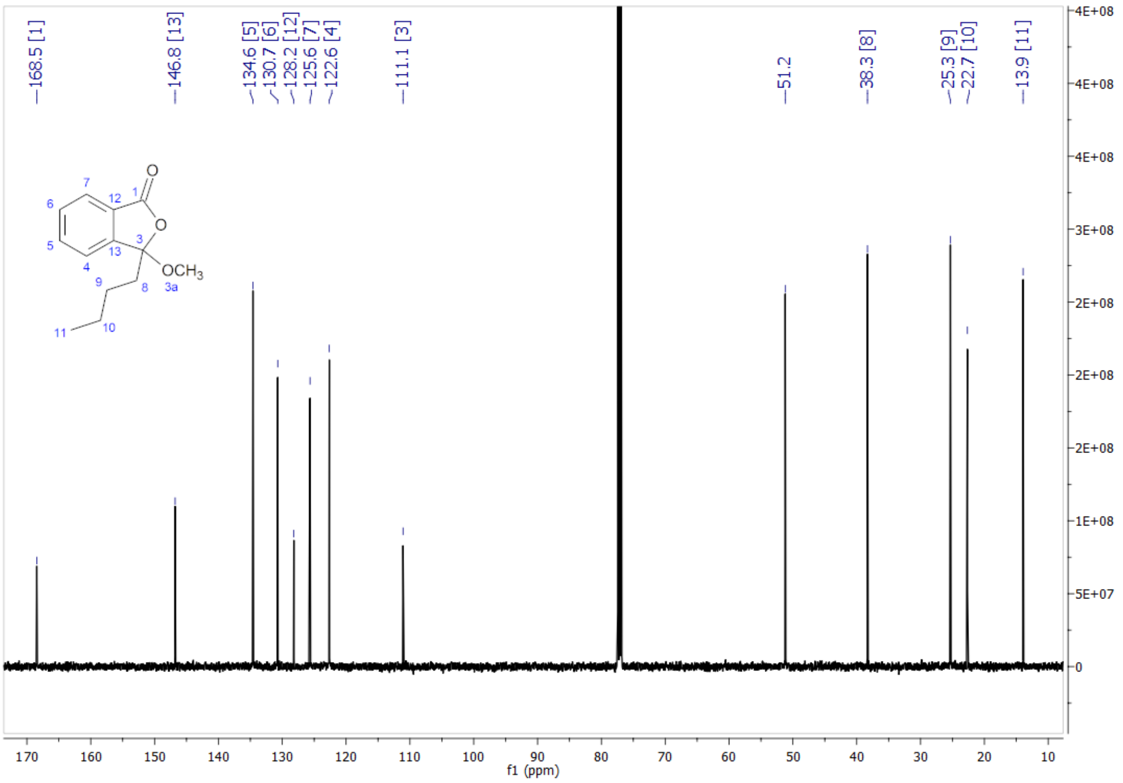

Figure S9. ^{13}C NMR spectrum of 3-butyl-3-methoxyphthalide (**3**).

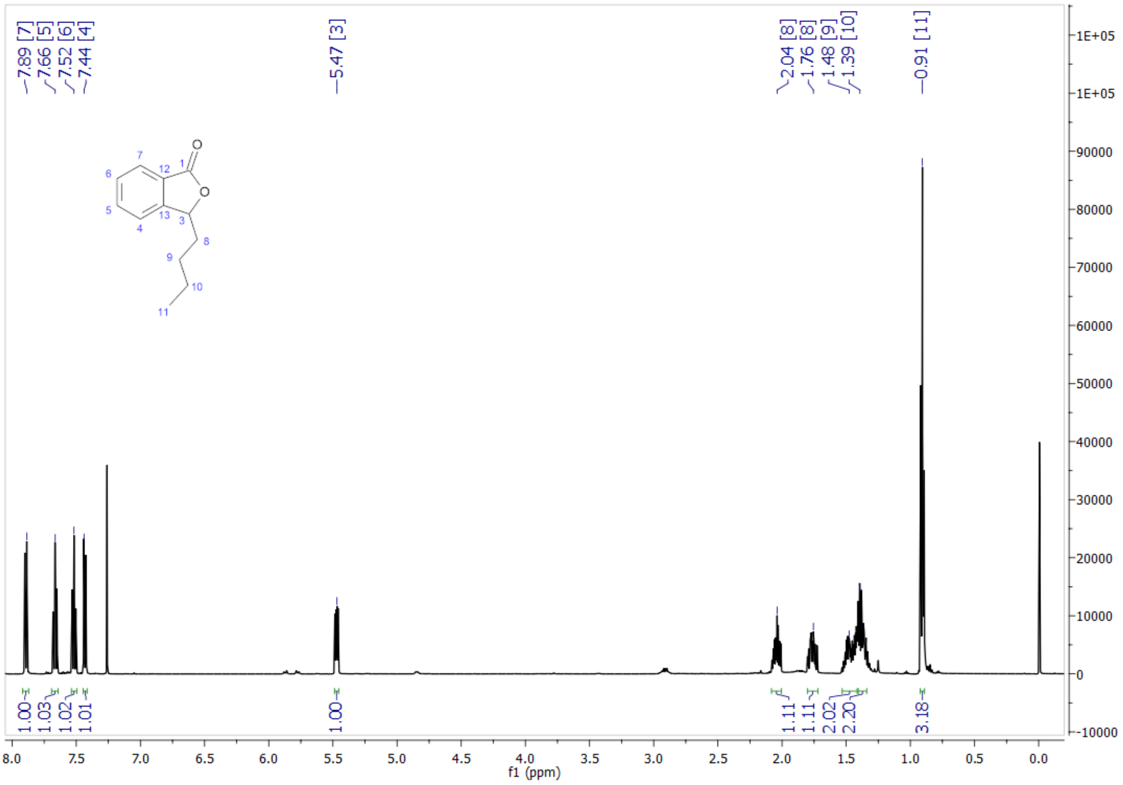

Figure S10. ^{1}H NMR spectrum of 3-*n*-butylphthalide (**4**).

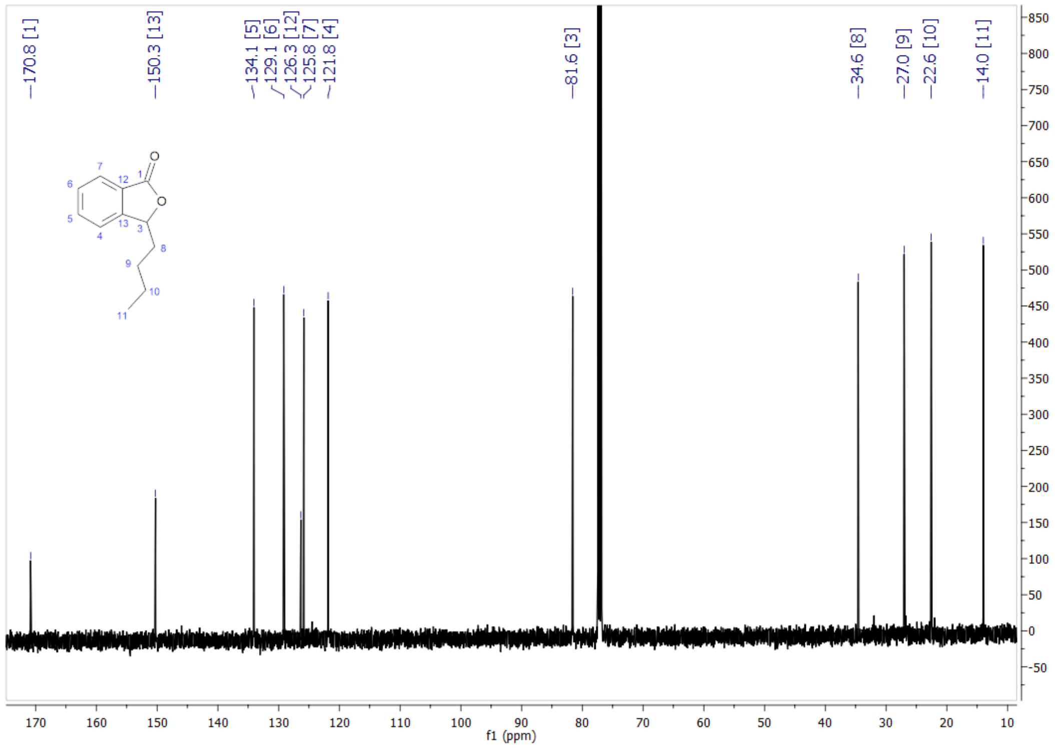

Figure S11. ^{13}C NMR spectrum of 3-*n*-butylphthalide (**4**).

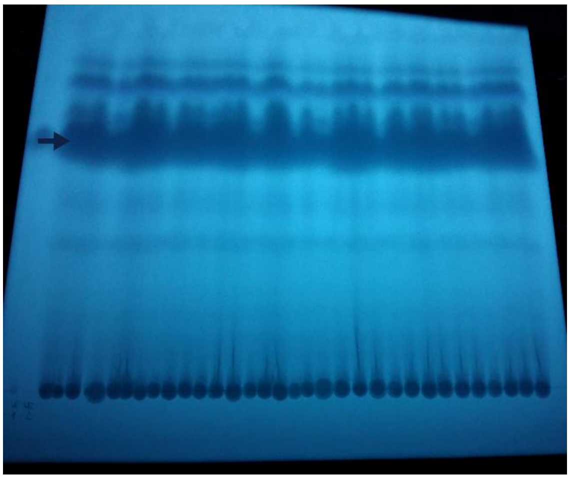

Figure S12. Purification of the product formed during biotransformation on reversed-phase thin layer chromatography plate. The product is indicated by an arrow.

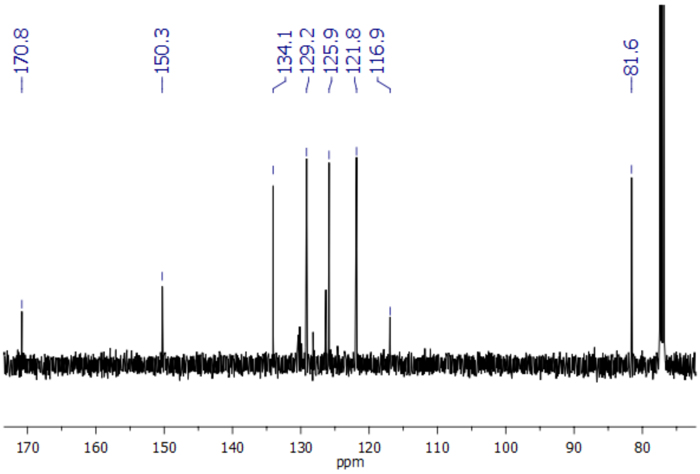

Figure S13. Fragment of the ^{13}C NMR spectrum of the *Chaetomium indicum* AM 158 biotransformation mixture.

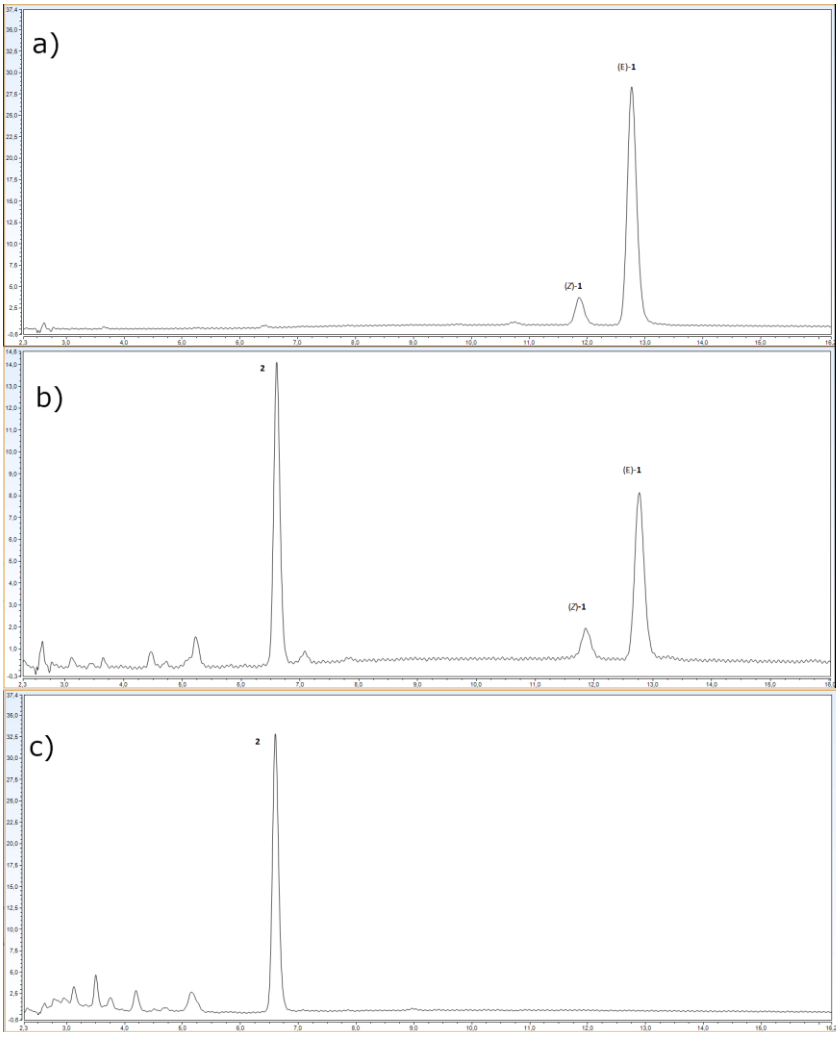

Figure S14. Chromatograms of 3-*n*-butylidenephthalide (**1**) – (*Z*) and (*E*) isomers (a) and the biotransformation mixtures of *Aspergillus candidus* AM 386 on days 2 (b) and 14 of the process (c).

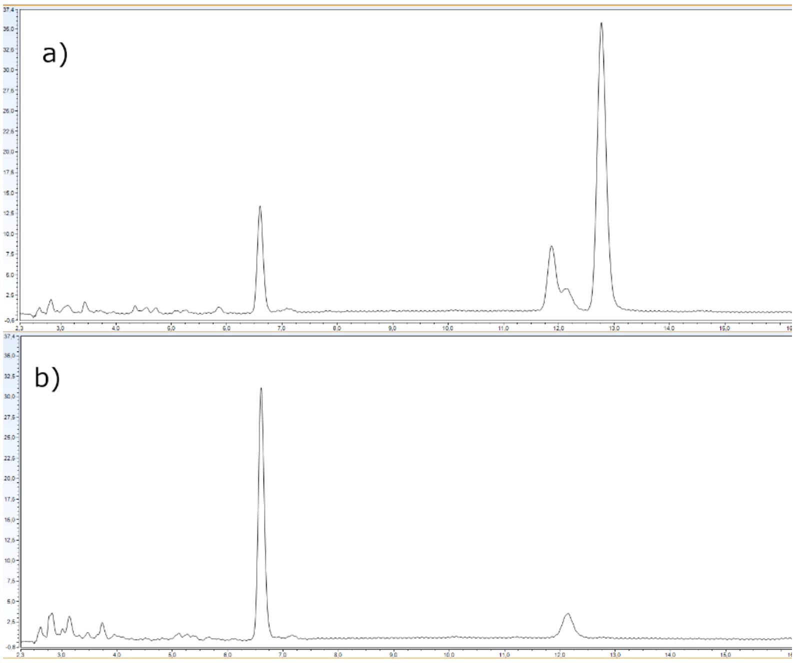

Figure S15. Chromatograms of the biotransformation mixtures of *Chaetomium indicum* AM 158 on days 2 (a) and 14 of the process (b).

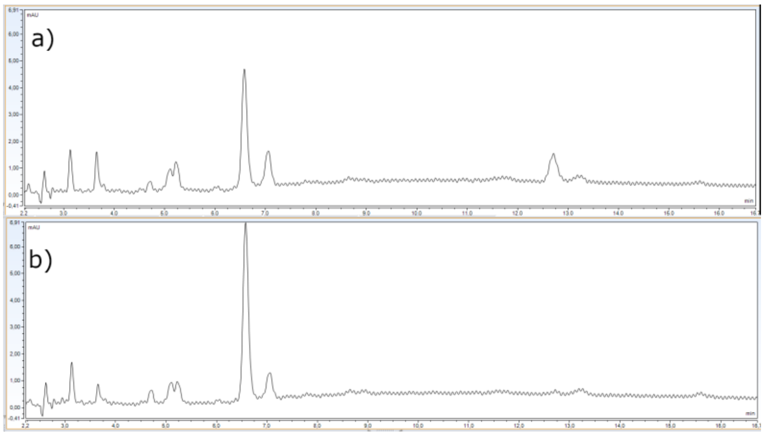

Figure S16. Chromatograms of the biotransformation mixtures of *Absidia cylindrospora* AM 336 on days 2 (a) and 14 of the process (b).
